# Education Research: Diversity in Neurology Graduate Medical Education Leadership

**DOI:** 10.1212/NE9.0000000000200060

**Published:** 2023-03-23

**Authors:** Morgan C. Jordan, Zahari N. Tchopev, Jeffrey C. McClean

**Affiliations:** From the Department of Neurology, Brooke Army Medical Center, San Antonio, TX.

## Abstract

**Background and Objectives:**

To describe the current landscape of gender and racial diversity among adult neurology residency and fellowship program directors (PDs). Diversity in medicine affects the quality of care provided to a diverse patient population. There are efforts in nearly every field of medicine to increase the diversity of the physician workforce. While there has been improvement in some of the known disparities in medicine such as gender disparities, these disparities have persisted in more senior academic positions in medicine.

**Methods:**

A data set was generated by the Association of American Medical Colleges for the purpose of this study using a variety of sources. The data included deidentified, person-level variables, including self-reported gender, race, or ethnicity, and the type of program for all PDs of Accreditation Council for Graduate Medical Education–accredited residencies and fellowships. This retrospective descriptive survey study sought to (1) describe the current gender and race climate of neurology PDs and (2) identify groups that may be disproportionally underrepresented in these positions compared with those in other specialties and levels of medical training. Descriptive statistics and tests of nonrandom association were performed to address the objectives.

**Results:**

We found that 56.7% of residency PDs and 58% of fellowship PDs are male. The male to female ratio of PDs was similar to current neurology residents who are 53.4% male. There were significantly more female medical students (51.5%) compared with all other categories of academic rank other than neurology residency PDs (41.1%). Only 4.3% of residency PDs were Black and only 3.7% were Hispanic. There were no Black fellowship PDs, and 5.1% were Hispanic. There were significantly more non-White medical students and trainees compared with each PD group. The breakdown of gender and ethnic diversity of neurology PDs was similar to that of PDs from all residencies and fellowships.

**Discussion:**

While there are many barriers to achieving diversity in medicine, program leadership in graduate medical education may be one of them. This report describes the current landscape of diversity among PDs of residency and fellowship programs in the United States. This study shows one snapshot in time, which can be used as a baseline during this era of change.

## Introduction

Gender and ethnic diversity improve productivity and innovation and increase workforce engagement.^1-4^ Specifically, there is increasing recognition that diversity in the medical education system creates a better equipped physician workforce to care for a diverse patient population. White students who attended more racially diverse medical schools felt they were better prepared to care for patients from racially minoritized groups, and a diverse faculty engenders greater cultural competency and diversity among medical students and residents.^5-7^ Diversity in medicine affects the quality of care provided to a diverse patient population by improving access to care, improving physician-patient communication, and reducing bias in healthcare decision-making.^8^ For example, physicians from underrepresented backgrounds are more likely to work in underserved areas regardless of specialty.^9,10^ Furthermore, African American and Latino patients report greater patient satisfaction, compliance, and participation in clinical research when treated by physicians from diverse and underrepresented backgrounds.^11^

There are efforts in nearly every field of medicine to increase the diversity of the physician workforce. Over the past decade, medical school enrollment has demonstrated an increase in students from underrepresented groups. However, this change has not been reflected in the highest ranks of academic medicine and leadership positions, as demonstrated by the Association of American Medical Colleges (AAMC) data as of 2018.^12^ While women now represent half of the medical student population, their representation declines as academic rank increases. This gender gap at high-ranking positions has been reported in neurology.^6^

Demographic data of the physician workforce including undergraduate and graduate medical education (GME) leadership based on academic rank is published frequently by the AAMC. However, data on the demographics of GME leadership, specifically program directors (PDs), is sparse. PDs not only serve as role models and mentors but are uniquely positioned to influence program culture, lead recruitment of a diverse workforce, and ensure that trainees have competencies in areas such as implicit bias and cultural awareness to be able to provide equitable care to diverse patient populations.

The primary objective of this study was to describe the current landscape of gender and ethnic diversity among adult neurology residency and fellowship PDs. This article addresses the importance and impact of diversity, equity, and inclusion in neurology and briefly discusses potential barriers and strategies to achieving diversity.

## Methods

This is a cross-sectional descriptive study. This study was reviewed by the Human Research Protections Office at Brooke Army Medical Center and determined to meet criteria for research not involving humans, and therefore, informed consent was not required.

A data set was created at our request by the AAMC that provided demographic data on all residency and fellowship PDs of Accreditation Council for Graduate Medical Education (ACGME)–accredited programs for the academic year of 2020–2021. The data set was deidentified by the AAMC by assigning each PD a unique ID and included the following variables: name of specialty, race/ethnicity, and gender. Current PDs were determined by the GME track, which collects PD names and contact information. Demographic information for those individuals was collected from various other AAMC applications and data collections including the Electronic Residency Application Service and Faculty Roster. Prioritization is given to the most recent self-reported information. Data were pulled on October 29, 2020, and included only programs that were ACGME-accredited in 2019 and 2020 and participated in the Program Survey GME Track. The GME Track Program Survey has high participation rates, typically above 94% but may not include all ACGME-accredited programs. Fellowship programs that have a large applicant pool from specialties other than neurology, such as sleep medicine and pain medicine, were not included. Programs that primarily serve pediatric patients, such as neurodevelopmental disabilities, were excluded because the scope of this study was limited to adult neurology.

Demographic data on current medical students and residents were obtained from publicly available reports published by the AAMC and cited where appropriate. These data were used for comparison with our new data set.

The various ways an individual may self-identify should be noted as a potential confounder when interpreting pooled demographic data from multiple different sources. The AAMC notes that the methodology for collecting race and ethnicity information for their databases has changed over time and may affect the consistency of findings. eAppendix 1 (links.lww.com/NE9/A21) further describes their change in methodology. Although race and ethnicity are not the same, data collected by the AAMC includes a single value for both. For the purposes of this study, race and ethnicity were defined as mutually exclusive groups categorized as one of the following: (1) Hispanic, (2) Black, (3) White, (4) Asian/Pacific Islander, (5) American Indian or Alaskan Native, (6) multiple/other, (7) unknown. For consistency and clarity, we will refer to race and ethnicity as race throughout the remainder of this article. eAppendix 2 further details how various subcategories were grouped into one of these larger categories. Gender demographic data are listed as (1) male, (2) female, (3) unknown, or, in some data sources, (4) nonbinary. Although various sources were used to create the data set, all data for race and gender used in this study were self-reported.

Data were analyzed using IBM SPSS Statistics. Descriptive statistics were performed for all programs within each group. The Chi-squared analysis was used to evaluate any effect of gender or race. Those listed as unknowns were considered nonrespondents and therefore not included in this analysis. For race subanalysis, individuals were considered “Non-White” if they provided any response other than White. A *p* value of less than 0.05 was used to infer statistical significance, but a Bonferroni correction was applied to counteract the multiple comparisons between the different groups. There were 10 gender comparisons, so an adjusted α level of 0.005 was applied, and similarly, there were 10 race comparisons, so an adjusted α level of 0.005 was applied.

### Data Availability

Anonymized data not published within this article will be made available by request from any qualified investigator.

## Results

Demographic information was collected on 12,443 PDs, of whom 5,295 were residency PDs, with the rest being fellowship PDs. There were 164 neurology residency PDs and 336 neurology fellowship PDs.

### Gender Diversity

**Figure 1:**
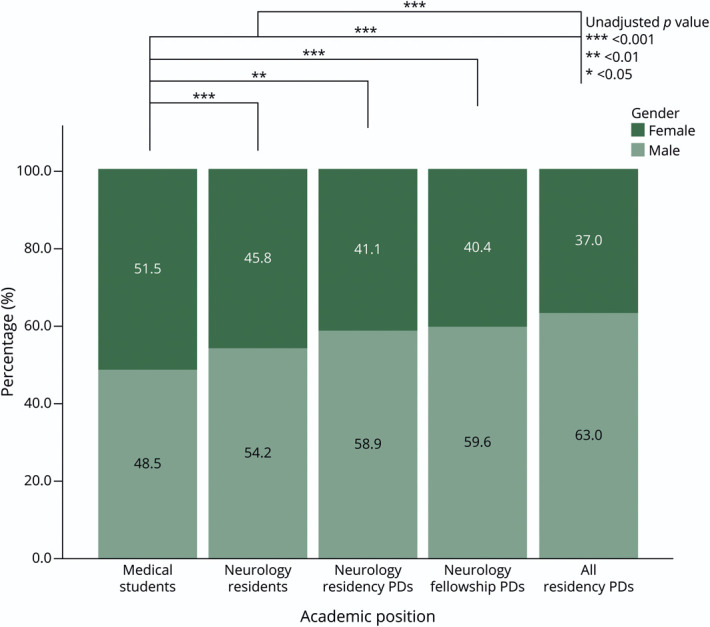
Percentages of Female vs Male Respondents at Each Level of Academic Position in Medicine PD = program director.

Table 1 reviews demographics between medical students, neurology residents, neurology residency PDs, neurology fellowship PDs, and all other PDs. We found that 56.7% of neurology residency PDs and 58% of neurology fellowship PDs were male. This slight male predominance was similar to the demographics of current neurology residents, which were 53.4% male. There was no difference in the gender breakdown of neurology residency PDs compared with that in all residency PDs or neurology fellowship PDs compared with that in all fellowship PDs (*p* = 0.29 and = 0.22, respectively). Figure 1 is a graphical representation of the gender percentages of each level of academic position as well as any predefined, unadjusted significant *p* values between each group, excluding unknowns. There was a significant difference in gender proportions between medical students (51.5% female) and all other categories of academic rank after Bonferroni correction (comparisons = 10, adjusted α level = 0.005) except for neurology PDs (41.1% female) (χ^2^ = 6.80, unadjusted *p* value = 0.009, *df* = 1). The Bonferroni-corrected gender proportion differences persisted between medical students and neurology residents (45.8% female) (χ^2^ = 38.14, unadjusted *p* value <0.001, *df* = 1), neurology fellowship PDs (40.4% female) (χ^2^ = 16.21, unadjusted *p* value <0.001, *df* = 1), and compared with all other residency PDs (37.0% female) (χ^2^ = 405.00, unadjusted *p* value <0.001, *df* = 1). Statistical analysis for between-group differences of neurology fellowship PDs was limited due to multiple subgroups either having a sample of less than 5 or a large proportion of unknowns, but is nonetheless presented.

**Table 1:** Gender and Race of Current Medical Students, Neurology Residents, Neurology Program Directors and All Residency Program Directors

	Medical students (n = 94,243), n (%)	Neurology residents (n = 3,062), n (%)	Neurology residency program directors (n = 164), n (%)	Neurology fellowship program directors (n = 336), n (%)	All residency program directors (n = 5,295), n (%)
Gender					
Female	48,530 (51.5)	1,381 (45.1)	65 (39.6)	132 (39.3)	1,865 (35.2)
Male	45,675 (48.5)	1,634 (53.4)	93 (56.7)	93 (58)	3,179 (60.0)
Nonbinary	^a^	0 (0)	^a^	^a^	^a^
Unknown	38 (0.04)	47 (1.5)	6 (3.7)	9 (2.7)	251 (4.7)
Race					
White	45,738 (48.5)	1,295 (42.3)	85 (51.8)	150 (44.6)	3,017 (57.0)
Black	7,126 (7.6)	127 (4.1)	7 (4.3)	0 (0.0)	196 (3.7)
Hispanic	6,295 (6.7)	199 (6.5)	6 (3.7)	17 (5.1)	154 (2.9)
Asian/Pacific Islander	21,586 (22.9)	687 (22.4)	25 (15.2)	79 (23.5)	656 (12.4)
American Indian or Alaska native	183 (0.2)	^a^	^a^	^a^	^a^
Multiple/other	11,083 (11.8)	271 (8.9)	8 (4.9)	30 (8.9)	161 (3.0)
Unknown	2,232 (2.4)	483 (15.8)	33 (20.1)	60 (17.9)	1,092 (20.6)

Abbreviation: AAMC = Association of American Medical Colleges.

Demographic information on current medical students was obtained from the AAMC for the academic year of 2020–2021.^13^ Demographic information on current neurology residents was obtained from the AAMC for the academic year of 2019–2020.^14^ Data for residency and fellowship program directors were obtained from our data set, which was abstracted for the academic year 2020–2021.

a Survey did not include this option to participants.

### Racial Diversity

**Figure 2:**
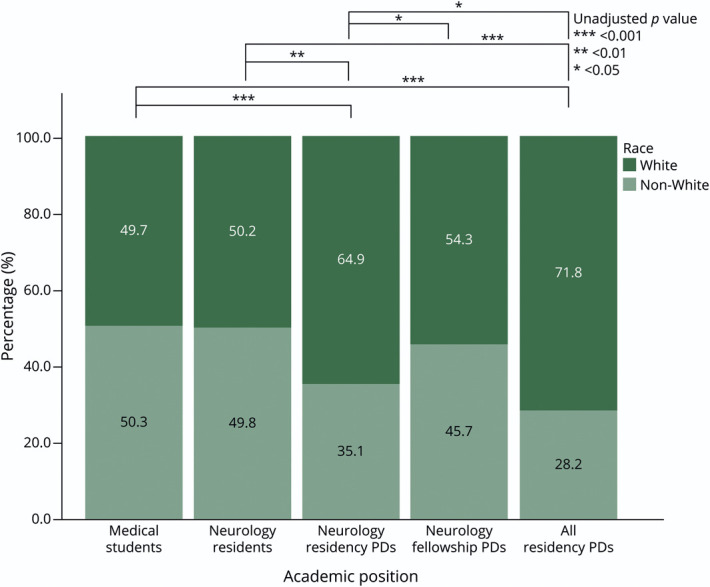
Percentages of White vs a Cumulative “Non-White” of Respondents at Each Level of Academic Position in Medicine PD = program director.

**Figure 3:**
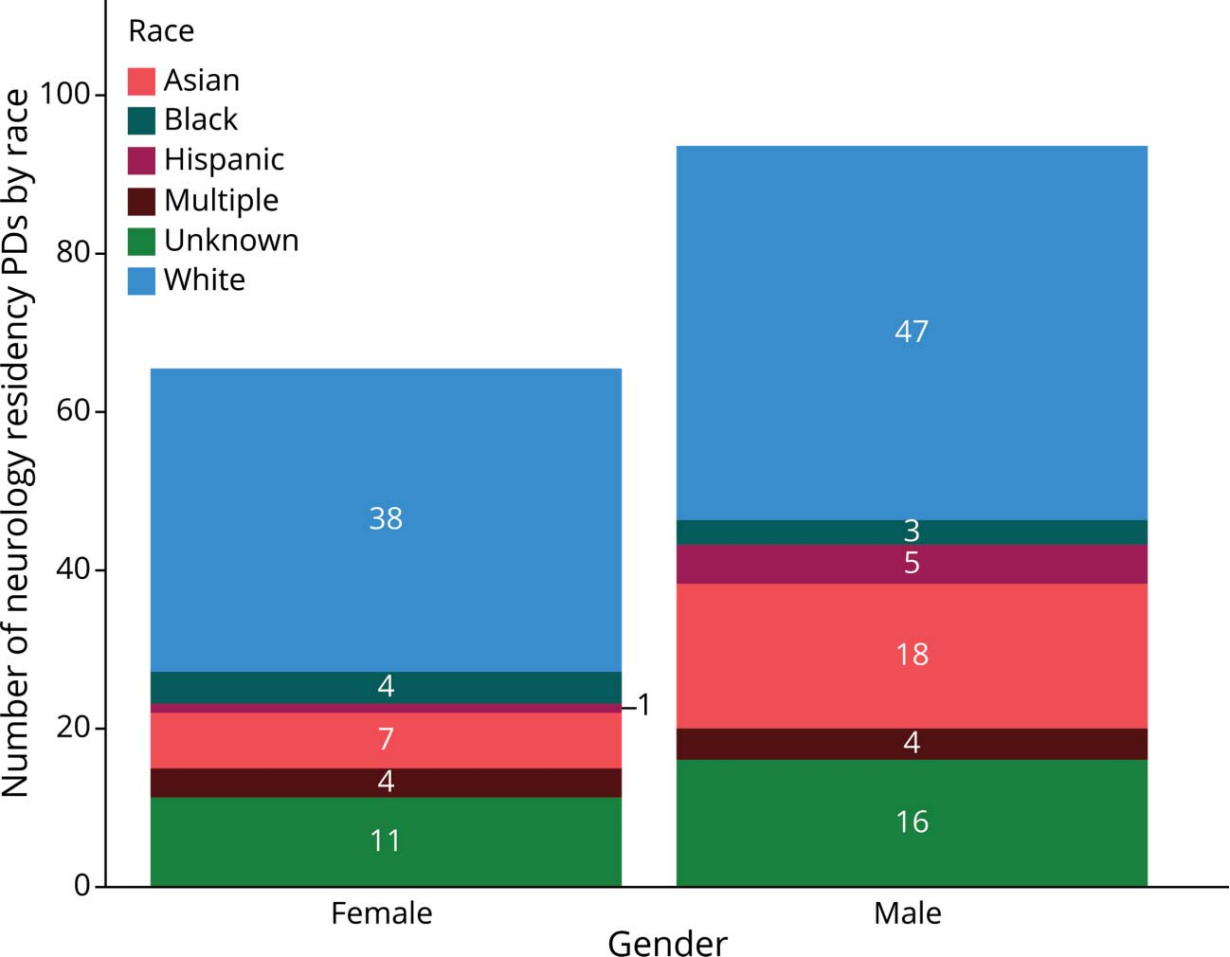
Number of Neurology Residency PDs Identifying as a Specific Race Stratified by Gender PD = program director.

**Figure 4:**
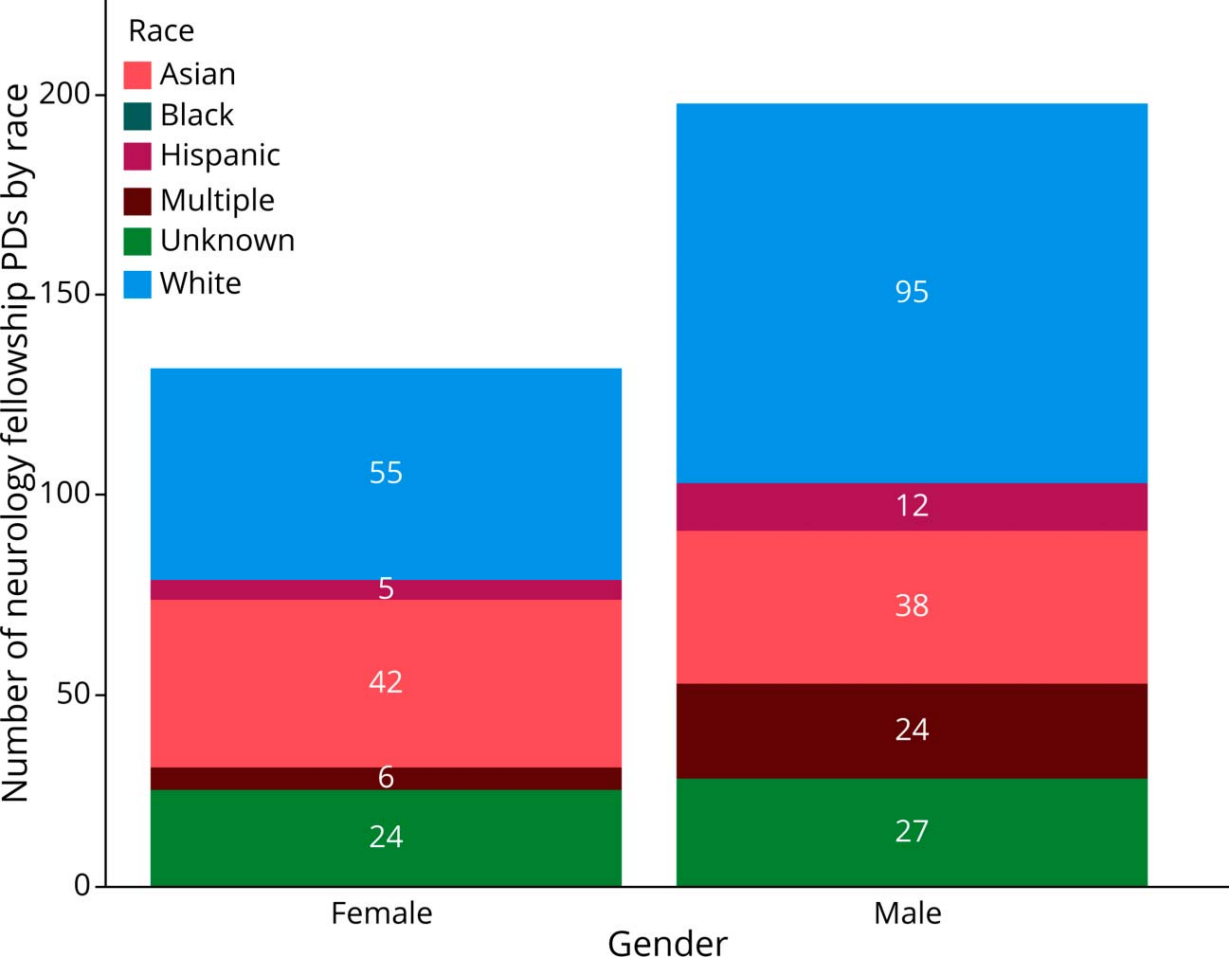
Number of Neurology Fellowship PDs Identifying as a Specific Race Stratified by Gender PD = program director.

There was no significant difference in the race of neurology residency PDs compared with residency PDs across all specialties. Only 4.3% of all neurology residency PDs were Black, and only 3.7% were Hispanic. There were no Black neurology fellowship PDs, and 5.1% were Hispanic. Statistical analysis was limited by the small or zero sample size of some of the ethnic subspecialty groups; therefore, analysis included comparing those identifying as White or Non-White (all other response choices, except for unknowns). There was a significant difference in race proportions after Bonferroni correction (comparisons = 10, adjusted α level = 0.005) between medical students (49.7% White) and neurology PDs (64.9% White) (χ^2^ = 12.05, unadjusted *p* value <0.001, *df* = 1), medical students and all PDs (71.8% White) (χ^2^ = 783.45, unadjusted *p* value <0.001, *df* = 1), neurology residents (50.2% White) and neurology PDs (64.9% White) (χ^2^ = 10.74, unadjusted *p* value <0.01, *df* = 1), and neurology residents and all residency PDs (71.8% White) (χ^2^ = 167.54, unadjusted *p* value <0.001, *df* = 1). This is represented graphically in Figure 2. Among neurology residency PDs, there was no association between gender and identifying as White or non-White (χ^2^ = 0.966; *df* = 1; *p* = 0.33). Similarly, among neurology fellowship PDs, there was no association between gender and identifying as White or non-White (χ^2^ = 1.471; *df* = 1; *p* = 0.23). Figures 3 and 4 depict the numbers of neurology residency and fellowship PDs, respectively, identifying as a specific race stratified by gender. The gender and race of neurology fellowship PDs was further broken down per subspecialty, as listed in Table 2.

**Table 2:** Gender and Race of Current Neurology Fellowship Program Directors by Subspecialty for the Academic Year 2020–2021

	Brain injury medicine (n = 1), n (%)	Clinical neurophysiology (n = 92), n (%)	Endovascular surgical neuroradiology (n = 4), n (%)	Epilepsy (n = 81), n (%)	Neuromuscular (n = 53), n (%)	Vascular (n = 105), n (%)
Gender						
Female	1 (100)	31 (33.7)	0 (0)	42 (43.4)	23 (43.4)	35 (33.3)
Male	0 (0)	57 (62.0)	4 (100)	36 (44.4)	29 (54.7)	69 (65.7)
Nonbinary	^a^	^a^	^a^	^a^	^a^	^a^
Unknown	0 (0)	4 (4.3)	0 (0)	3 (3.7)	1 (1.9)	1 (1.0)
Race						
White	^a^	39 (42.4)	1 (25.0)	39 (48.1)	26 (49.1)	45 (42.9)
Black	^a^	^a^	^a^	^a^	^a^	^a^
Hispanic	^a^	6 (6.5)	1 (25.0)	2 (2.5)	3 (5.7)	5 (4.8)
Asian/Pacific Islander	^a^	21 (22.8)	1 (25.0)	21 (25.9)	13 (24.5)	22 (21.9)
American Indian or Alaska native	^a^	^a^	^a^	^a^	^a^	^a^
Multiple/other	^a^	7 (7.6)	1 (25.0)	4 (4.9)	6 (11.3)	12 (11.4)
Unknown	1 (100)	19 (20.7)	^a^	15 (18.5)	5 (9.4)	20 (19.0)

a Survey did not collect or report this option.

## Discussion

Recruitment of students who are underrepresented in medicine (URM) into the field of neurology or into various neurologic subspecialties is limited by the number of medical students and current residents representing these groups. As the number of medical students from diverse backgrounds increases, it is imperative that neurology GME leaders continue to recruit those who URM into the field of neurology. If the leadership of GME programs lack diversity, it appears less likely that a diverse trainee population will be recruited and trained, hindering diversity of future leaders by limiting the pool of diverse applicants in a repetitive cycle. This pattern has been shown in recruiting medical students but is also reflected in the finding that diverse companies are better at recruiting and retaining a diverse workforce.^1,4,15^ Our review further supports this pattern, with a significantly greater proportion of female medical students than neurology residents or PDs, and then the proportion remains stagnant from female neurology residents to PDs. A similar finding was noted when looking at the gender of neurology fellowship PDs compared with their potential applicants from neurology residency. In addition, there is a larger representation of Black and Hispanic medical students than Black and Hispanic PDs but with a similar breakdown of current Black and Hispanic residents as residency PDs. As the medical student population continues to diversify, conscious effort must be made to ensure that there is an inclusive culture at individual programs to allow for opportunities for leadership positions to continue to similarly diversify.

This study shows that while the gender gap among PDs is small, there remains little representation from most ethnic groups. Our data are similar to the 2018 AAMC Diversity in Medicine statistics, which showed only 3.6% of full-time faculty at US medical schools were Black and only 5.5% were Hispanic,^16^ and the 2017 American Academy of Neurology (AAN) PD survey study also found similarly low rates of Black and Hispanic residency PDs.^17^ A national survey in 2020 of neurology vice chairs for education at GME programs showed similar demographic data for these leaders with 51% identifying as male and 85% White.^18^ The 2017 AAN PD survey did show a higher proportion of men in PD roles (61%) compared with our data (57.6%).^17^ There have been increases in the proportion of faculty positions held by individuals who are URM in the last 12 years. However, at the current rate of this increase in neurology, it will take 40 years for there to be an equitable representation of race at these high-level positions.^8^

Although nearly all medical schools and graduate medical training programs have incorporated initiatives to increase diversity, some areas of medical education and leadership are slower to show results, as measured by the current status of diversity in program leadership. However, this study does not measure inclusivity, which is harder to objectively measure but is still a goal worth pursuing when it comes to creating an environment where all backgrounds are able to achieve the highest ranks of leadership.

There have been many proposed barriers and solutions to promote diversity, and the approach must be multifactorial. Lack of those who are URM in these positions of power limit the number of role models or mentors, which then limits the opportunity for knowledge sharing about how to succeed in academic medicine and sponsorship and limits the size of formal and informal professional networks. Lack of others who are URM in leadership positions has been cited as a barrier for those who are URM in other fields including pediatrics, obstetrics and gynecology, and orthopedics.^19-22^ Similarly, affinity bias, which is a natural tendency and often unconscious preference for people who are similar to them, can affect opportunities for selection for leadership positions when there is a lack of diversity in the current leadership pool. This study further supports the argument that diverse leadership and role models are likely to encourage individuals who are URM to pursue a certain field of medicine. Another potential barrier is the differences in financial need of persons from minoritized groups, which may steer them away from academic medicine and toward the generally more lucrative private practice. The slower climb of the academic ranks for those physician URM may further cement this process. One commonly cited obstacle facing individuals who are URM is the idea of diversity tax. The diversity tax refers to the tendency to place an unintentional burden on marginalized individuals to address diversity, equity, and inclusion. This has the potential implications of not only leading to burnout of those individuals but also limits their opportunity to dedicate their time and expertise to other important leadership opportunities. Many strategies focus on the pipeline of available applicants and recruiting efforts to continue the trend of increased representation of diverse backgrounds of medical student applicants. Based on the available data showing an increasing number of medical students who are URM, these efforts have been successful and should continue to be targeted. One limitation of this strategy is that there is a lag time before the effects of these efforts are seen due to the length of the training pipeline for students; however, there are additional strategies that can target the current physician pool. There are societal and cultural obstacles that certainly affect diversity efforts such as implicit bias. Implicit bias has been shown to be present in physicians at the same rate of the general population and correlates with quality of care.^23,24^ These biases are deep rooted and unconscious and require conscious effort to avoid allowing them to affect decision-making. Implicit biases held by selection committees or departmental leadership have the potential to cause capable physicians from minoritized or marginalized backgrounds to be inadvertently overlooked during the selection process for these leadership positions. Implicit bias and cultural competency training are crucial to reducing disparities and inequalities and have the potential to effect change more rapidly because you can target the current workforce, which eliminates that concern for the potential time lag from training and implementation of equity, diversity, and inclusion solutions if only targeting recruitment.

The lack of diversity of PDs is not unique to neurology GME leadership but is seen in all of academic medicine, as demonstrated by the AAMC diversity data.^16,17^ There are likely many barriers that physicians from underrepresented groups face when ascending the ranks of academic hierarchy, and it should be noted that this is not an exhaustive list. In this study, we focus on exploring the potential impact that this lack of diversity can have on recruiting a diverse workforce into the field of neurology. Important next steps should include identifying and addressing all the barriers to diversity that neurologists face in academic medicine.

While this study only analyzed 1 snapshot in time, it is clear that gender and race are still predictive of a physician's access to opportunities and future career outcomes.

There are several limitations of this study. As previously mentioned, race and ethnicity are distinct but have been conflated in the data used for this analysis. The collected data also largely treat gender as binary, so it cannot accurately depict the gender identity of all studied individuals. In addition, persons who identify as lesbian, gay, bisexual, transgender, or queer may be underrepresented among PDs, but we lack the comprehensive data to determine this definitively. While this study focuses on gender and race, the need for diversity in medicine is much broader, and these efforts must be intentional in recognizing and recruiting individuals from all underrepresented and minoritized groups. This study was designed to outline only the current landscape of diversity of PDs and was not able to show trends in the diversity to be able to determine whether there is progress or not. Longitudinal data and additional data from other specialties would be helpful in evaluating these trends across time and specialties. Future studies gathering these data would add needed perspective to map the best course forward and ensure key stakeholders were engaged in addressing this gap. Another limitation of this study is that many neurology fellowships and disciplines are not accredited by the ACGME and therefore were not captured in this study. In addition, child neurology residencies and subspecialties were not studied. Last, looking only at the current diversity of the PDs does not necessarily capture whether the culture of a specialty or program is inclusive or supportive of diversity.

With increasing diversity of the general population in the United States, the need for a diverse and culturally competent healthcare workforce to provide equitable care is paramount. While there are many barriers to achieving diversity in medicine, program leadership in GME may be one of them. A conscious effort must be made to continue the environment of inclusivity so that this increase in diversity seen in the trainee pipeline may continue to expand into the leadership roles of academic medicine. This study demonstrates one snapshot in time, which can be a useful baseline from which to plan deliberately and track progress in pursuit of a diverse group of neurologists better able to provide care to the increasingly diverse patient population.

## Appendix Authors

**Table:**

Name	Location	Contribution
Morgan C. Jordan, DO	Department of Neurology, Brooke Army Medical Center, San Antonio, TX	Drafting/revision of the article for content, including medical writing for content; major role in the acquisition of data; study concept or design; and analysis or interpretation of data
Zahari N. Tchopev, MD, MBA	Department of Neurology, Brooke Army Medical Center, San Antonio, TX	Drafting/revision of the article for content, including medical writing for content; analysis or interpretation of data
Jeffrey C. McClean II, MD	Department of Neurology, Brooke Army Medical Center, San Antonio, TX	Drafting/revision of the article for content, study concept or design and major role in the acquisition of data

## Glossary

AAMC: Association of American Medical Colleges
AAN: American Academy of Neurology
ACGME: Accreditation Council for Graduate Medical Education
GME: graduate medical education
PD: program director
URM: underrepresented in medicine
